# Bioengineered cell therapies for pediatric solid tumors: unmet needs and a measurement-integrated approach

**DOI:** 10.1088/2516-1091/aea045

**Published:** 2026-09-11

**Authors:** Ana M Sandoval-Castellanos, Yu-Rim Ahn, Robert J Canter, Erin G Brown, Jinhwan Kim

**Affiliations:** 1 Department of Biomedical Engineering, University of California Davis, Davis, CA 95616, United States of America; 2 Department of Surgery, School of Medicine, University of California Davis, Sacramento, CA 95817, United States of America; 3 Department of Ophthalmology and Vision Science, University of California Davis, Sacramento, CA 95817, United States of America; 4 Center for Bioengineering in Medicine, School of Medicine, University of California Davis, Sacramento, CA 95817, United States of America

**Keywords:** pediatric solid tumor, cancer immunotherapy, CAR T, CAR NK, non-invasive imaging

## Abstract

Pediatric solid tumors continue to pose a major therapeutic challenge, with survival gains lagging behind those achieved in pediatric hematologic malignancies. While traditional approaches have focused on dose escalation and intensification of systemic therapies to improve survival, this strategy is often limited by significant short- and long-term morbidity from intensive multimodal treatment. Because children have developing organs and decades of life ahead, therapeutic strategies must balance durable tumor control with preservation of neurodevelopment, organ function, and quality of life. Immunotherapy has generated significant interest as an alternative to dose escalation; however, clinical translation in pediatric solid tumors has been limited by antigen heterogeneity, tumor plasticity, immune-cold or immune-excluded phenotypes, and a profoundly immunosuppressive tumor microenvironment. Bioengineered cellular therapies, particularly chimeric antigen receptor (CAR) T cells and CAR-modified natural killer (CAR NK) cells, provide a modular platform to address these barriers through synthetic receptor design, multi-antigen targeting, controlled activation, and improved trafficking to anatomically restricted sites such as the brain. However, engineering advances alone are unlikely to achieve durable benefit without parallel integration of quantitative *in vivo* monitoring capable of reporting biodistribution, persistence, and functional engagement. Non-invasive imaging and measurement-enabled approaches can provide mechanistic insight into therapeutic performance, discriminate between delivery failure and functional dysfunction, and support rational iteration of construct design, dosing, and route of administration. In this perspective, we review the current status of CAR T and CAR NK therapies in pediatric solid malignancies, outline key biological and engineering challenges, and propose a pediatric-centered development framework that integrates controllable cell engineering with quantitative, non-invasive assessment of *in vivo* behavior. By embedding measurement into the therapeutic design loop and prioritizing long-term safety and developmental outcomes alongside efficacy, next-generation engineered cell therapies may evolve toward adaptable, precision-guided systems capable of improving both survival and quality of life for children.

## 1. Introduction

Although pediatric hematologic malignancies have seen transformative advances, outcomes in high-risk pediatric solid tumors have improved only modestly, and for many entities have remained largely unchanged over the past two decades [1–3]. Central nervous system (CNS) are the most common solid tumor in children with CNS tumors accounting for roughly 20% of newly diagnosed pediatric cancers; non-CNS solid tumors, also known as extracranial solid tumors, include neuroblastoma, sarcomas, nephroblastoma, and hepatoblastoma [4]. Many of these solid tumors are notoriously difficult to treat, largely due to the metastatic disease and the development of treatment resistance. Furthermore, treatment is complicated by smaller blood volumes and developing organs of pediatric patients, increasing the risk of toxicity and potential for late treatment effects, posing fundamental challenges in the treatment of pediatric patients with cancer [5]. Accordingly, the primary clinical objective is not only durable tumor control, but durable tumor control with minimized long-term morbidity.

Most aggressive pediatric solid tumors are treated with a multimodal approach including high-dose chemotherapy, radiation, and surgery, and the conventional approach has been to escalate systemic treatments to improve outcomes. This treatment paradigm has improved survival in some settings, but also comes at the cost of substantial acute toxicities from the intensive treatment and late side effects, including neurocognitive, endocrine, cardiovascular, and psychosocial sequelae [6–8]. By early to mid-adulthood, about 40% of childhood cancer survivors experience severe or life-threatening chronic health conditions, underscoring that dose intensification alone has limits in achieving durable cures with acceptable toxicity [9]. These realities highlight the need for therapies that can deliver antitumor potency without compounding irreversible developmental injury.

Cell-based cancer immunotherapy has transformed pediatric hematologic oncology, but solid tumors pose a different immunobiological problem [10, 11]. Unlike leukemia, where abundant circulating targets and lineage-restricted antigens enable consistent immune engagement, pediatric solid tumors often present mosaic and therapy-evolving antigen expression, creating immediate vulnerability to antigen-low escape [12–15]. At the same time, these tumors are commonly immune-cold or immune-excluded, with suppressive myeloid/Treg niches and inhibitory cues that blunt effector function after tumor entry. Here, immune-cold refers to tumors with limited baseline immune-cell infiltration, whereas immune-excluded refers to tumors in which immune cells are present but spatially restricted from effective entry into tumor regions. In the CNS, anatomical constraints compound these barriers by limiting systemic trafficking and making radiographic interpretation difficult when inflammation can mimic progression [16, 17]. These challenges are amplified in pediatrics, where low-level on-target/off-tumor recognition can have outsized developmental consequences and where the acceptable threshold for neurotoxicity and prolonged immune perturbation is lower [17]. As a result, immunotherapies that are highly effective in hematologic disease can yield inconsistent durability in pediatric solid tumors, and conventional endpoints provide limited mechanistic insight into whether failure reflects delivery, targeting, or functional suppression. Although we examine these pediatric-specific constraints in detail in the sections that follow, it is important to recognize at the outset that pediatric solid tumors are not simply smaller versions of adult disease: their target antigens are frequently developmentally regulated and shared with normal growing tissues, organ reserve is limited, and the relevant safety horizon spans decades rather than years, so that pediatric requirements differ qualitatively from those in adults [18]. Pediatric sarcomas, including Ewing sarcoma and rhabdomyosarcoma, illustrate this convergence, combining heterogeneous surface-antigen expression, dense stroma that limits effector-cell trafficking, and immunosuppressive microenvironments with a narrow tolerance for off-tumor recognition of developmentally expressed antigens [19–21].

**Figure 1.**
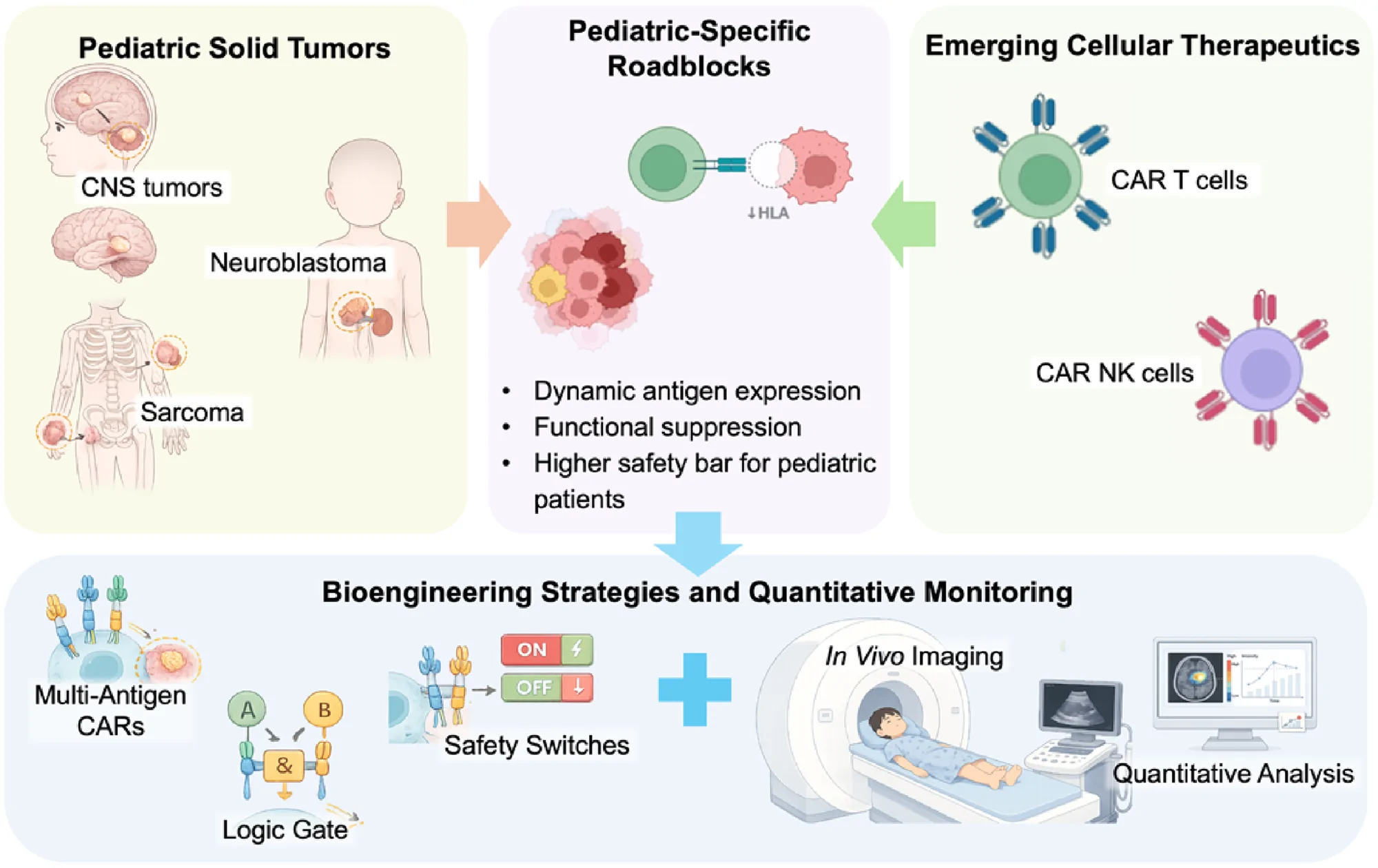
Schematic illustration of a pediatric-centered framework for bioengineered cell therapy development. It highlights pediatric-specific roadblocks and bioengineering strategies, integrated with quantitative imaging, for the development of next-generation cell-based therapies for pediatric cancer. Created in https://BioRender.com.

In this perspective article, we focus on bioengineered cellular therapies for pediatric solid tumors, primarily chimeric antigen receptor (CAR)-transduced T (CAR T) cells and CAR-transduced natural killer (CAR NK) cells, and argue for a pediatric-first development logic. Throughout this article, we use ‘engineered cell therapies’ to refer primarily to biologically or chemically modified cellular platforms, including CAR T and CAR NK cells, whereas ‘cell-based therapies’ is used more broadly to include both engineered and non-engineered adoptive cellular approaches. We discuss representative preclinical and clinical studies to identify recurring biological and translational barriers in engineered cell therapies for pediatric solid tumors. We use these examples to guide a discussion of how cell therapies can be designed specifically for pediatric tumors and how their efficacy can be quantitatively assessed *in vivo*. Specifically, we propose that progress will depend less on incremental receptor optimization alone and more on an integrated design-and-evaluate loop that combines 1) engineering strategies that tolerate antigen diversity, resist suppressive microenvironments, and incorporate controllability, with 2) quantitative *in vivo* monitoring to measure biodistribution, persistence, and functional engagement over time. We first summarize the current status and limitations of CAR T and CAR NK cell approaches in pediatric solid tumors. We then highlight key roadblocks, including antigen heterogeneity, immune exclusion, and an immunosuppressive tumor microenvironment (TME), and discuss emerging engineering solutions aimed at overcoming these barriers. Finally, we outline future directions that prioritize low toxicity and incorporate pediatric patient-appropriate assessment metrics, with the goal of recalibrating how efficacy and safety are balanced in children (figure 1).

## 2. The emergence of cellular therapies: CAR T cells and CAR NK cells

In 2017, the U.S. Food and Drug Administration approved the first CD19 CAR T cell therapy for pediatric and young adult patients with acute B lymphoblastic leukemia (ALL). CAR T cells are generated by collecting a patient’s T cells and genetically engineering them to express a synthetic receptor that recognizes a tumor-associated surface antigen and converts that binding into T cell activation and cytotoxicity [22, 23]. This engineered receptor bypasses conventional major histocompatibility complex-restricted antigen presentation, enabling potent, antigen-specific killing upon engagement of the target [24]. After ex vivo expansion, the modified cells are reinfused to provide a living, self-amplifying therapeutic that can persist and mediate sustained immune pressure [22–24]. This milestone marked a turning point in cancer immunotherapy, paving the way for the approvals of additional CAR T cell therapies targeting other hematologic malignancies in the years that followed [25–27]. Furthermore, this success has naturally prompted efforts to extend CAR T cell therapy to pediatric solid tumors, particularly neuroblastoma and high-grade CNS tumors. Early GD2-targeted CAR T trials in neuroblastoma [28, 29] and B7–H3-targeted CAR T therapy in pediatric CNS tumors [30] established the feasibility and repeated administration of CAR T cells to children, with clear signals of antitumor activity but also frequent relapse and heterogeneous responses [31–33]. For example, a phase I/II trial of GD2-CAR T in neuroblastoma reported an overall response rate of 63%, including nine complete responses, with a safety switch successfully activated in a child who developed severe toxicity [28]. In children, intraventricular (ICV) B7–H3 CAR T has enabled repeat intracranial dosing in the outpatient setting with procedure-level safety; in BrainChild-03 (diffuse intrinsic pontine glioma, DIPG), 21 children received 253 ICV doses (median 9 per patient; range 1–81), no immune effector cell–associated neurotoxicity syndrome (ICANS) was observed, grade ⩾3 neurologic events were infrequent (e.g. hydrocephalus 1/21, ∼5%), and median survival from diagnosis was 19.8 months with three survivors at 44–52 months [30, 34]. In addition, GD2-directed CAR T cells delivered intravenously and then intracerebroventricularly produced radiographic and clinical responses in H3K27M-mutated diffuse midline glioma, including major volumetric regressions and one complete response sustained beyond thirty months [35, 36], and HER2-directed CAR T cells given after lymphodepletion were safe and associated with clinical benefit in high-risk sarcoma [37]. These examples demonstrate the potential of CAR T-based approaches in treating pediatric solid tumors. However, most ongoing trials remain early-phase and exploratory, leaving significant room for further improvements. Additional concerns include the astronomical cost of CAR T manufacturing and well-known life-threatening side effects, such as cytokine release syndrome (CRS), neurologic toxicities (e.g. ICANS), prolonged cytopenias, and more [38].

NK cell platforms offer complementary advantages. NK cells are cytotoxic innate lymphocytes that rapidly recognize stressed or malignant cells, secrete inflammatory cytokines, and induce tumor-cell apoptosis. An advantage of NK cells is that an autologous source is not necessary, and an allogeneic source (e.g. umbilical cord blood, stem cells) can be expanded and stored off the shelf, which can reduce manufacturing time and cost compared with T cell-based therapies. In pediatric programs, allogeneic CAR/NK workflows may mitigate time-to-infusion constraints, as autologous CAR T typically has an average vein-to-vein interval of ∼3–6 weeks, whereas allogeneic processes decouple manufacturing from patient scheduling and enable on-demand administration; however, pediatric solid tumor data remain limited [39, 40]. In addition, NK cell therapies have a generally favorable safety profile, with low rates of severe CRS, neurotoxicity or classical graft-versus host disease (GvHD) [41, 42], making them an attractive off-the-shelf alternative for immunotherapy accessible to all patients [26, 42, 43]. CAR NK cells retain their intrinsic NK-killing mechanisms, making them safer and lowering toxicity concerns. An early pediatric neuroblastoma study evaluating allogeneic NK cell line-based GD2-CAR NK infusion suggests antitumor activity with a favorable safety profile [44]. Within NK-lineage approaches in neuroblastoma, an interim phase-1 report of GD2-CAR NKT cells (*n* = 12) showed an objective response rate of 25% (CR 1, PR 2), no dose-limiting toxicities, one grade-2 CRS resolved with tocilizumab, and no maximum tolerated dose reached [45]. Although rare, serious events such as hyperleukocytosis have been reported in NK cell-based therapeutics in some other cancer models, such as B-cell malignancies [46] and non-small cell lung [47] cancer (NSCLC), thus further investigations into dosing and monitoring are necessary for pediatric application. Representative clinical trials are summarized in table 1.

**Table 1.** Representative clinical trials of engineered cell therapies in pediatric solid tumors. DIPG, diffuse intrinsic pontine glioma; DMG, diffuse midline glioma; ORR, objective response rate; CR, complete response; PR, partial response.

Target antigen	Cell platform	Target tumor	Performance	Toxicity	References
GD2	CAR T cells with iCasp9 safety switch	High-risk neuroblastoma; Phase 1/2; *n* = 54	ORR 66%, 5 year overall survival 42.7%	CRS was mostly mild; grade 3 ICANS occurred in 4 children and was controlled by activation of the iCasp9 safety switch	[28, 29]

GD2	CAR T cells	DIPG/spinal DMG; Phase 1; *n* = 4	Clinical and radiographic improvement in 75% of patients; 1 CR	Tumor location-associated neurotoxicity and high-grade CRS were observed; no on-target/off-tumor toxicity was reported	[35, 36]

B7–H3	CAR T cells	DIPG/other CNS tumors; Phase 1; *n* = 21	Median survival from diagnosis 19.8 months	No ICANS was observed; grade >3 neurologic events were infrequent	[30]

HER2	CAR T cells	Sarcoma; Phase 1; *n* = 14	CR 21%, stable disease 29%	Safe; no dose-limiting toxicities were observed at the studied dose	[37]

GD2	CAR NKT cells	Relapsed neuroblastoma; Phase 1; *n* = 12	OPR 25% including 1 CR and 2 PR	One case of grade 2 CRS occurred and resolved with tocilizumab	[45]

Because CAR T and CAR NK cells differ in persistence, expansion kinetics, manufacturing model, and safety profile, they should not be viewed as interchangeable engineered cell platforms. Instead, each platform presents a distinct translational trade-off for pediatric solid tumors: CAR T cells currently offer greater clinical maturity and potential for durable persistence, whereas CAR NK cells may provide advantages in safety, allogeneic manufacturing, repeat dosing, and scalability (table 2). Overall, CAR T cells may be preferable when sustained *in vivo* expansion and long-term immune surveillance are required, provided that toxicity can be managed. In contrast, CAR NK cells may be particularly attractive for pediatric indications in which lower inflammatory toxicity, off-the-shelf access, and repeat dosing are prioritized. However, the pediatric solid tumor evidence base remains substantially stronger for CAR T than for CAR NK therapy, and future studies will need to determine whether the theoretical safety and manufacturing advantages of CAR NK cells translate into durable clinical benefit in children.

**Table 2.** Key translational differences between CAR T and CAR NK cell platforms for pediatric solid tumors.

	CAR T cells	CAR NK cells	Notes
Clinical maturity	Greater clinical experience, including early-phase pediatric solid tumor trials.	Less clinically mature; pediatric solid tumor data remain limited.	CAR T currently has stronger clinical precedent, while CAR NK remains an emerging platform.

Persistence and expansion	Robust antigen-driven expansion and longer persistence.	Typically shorter persistence and more limited *in vivo* expansion; may require cytokine support or repeat dosing.	CAR T may support durable tumor control but increases risk of prolonged toxicity; CAR NK may offer more controllable exposure.

Safety	Higher risk of CRS, ICANS, prolonged cytopenias, and on-target/off-tumor toxicity.	Generally lower risk of severe CRS, ICANS, and GvHD, although safety depends on product design and cytokine support.	Lower inflammatory toxicity may be attractive in children, especially for CNS tumors.

Manufacturing	Often autologous, individualized, costly, and time-intensive.	Potentially allogeneic/off-the-shelf, scalable, and less costly per dose if batch production is achieved.	Off-the-shelf availability may benefit children with rapidly progressive disease or limited access to specialized centers.

Main translational trade-off	Greater persistence and clinical maturity, but higher toxicity and manufacturing burden.	Favorable safety and scalability, but shorter persistence and less pediatric clinical validation.	Platform choice should be case-specific than interchangeable.

Taken together, early phase studies with CAR T and CAR NK approaches in pediatric solid tumors demonstrate strong feasibility and utilizing engineered cell-based immunotherapies as a possible approach, not simply as ‘more therapy,’ offers specific relevance in pediatric oncology. However, durable benefit remains inconsistent, and toxicity, logistics, and access constraints remain substantial. The following section examines how pediatric tumor biology and developmental safety constraints translate into specific design requirements for next-generation cell therapies.

## 3. Navigating the roadblocks in pediatric solid tumor

**Figure 2.**
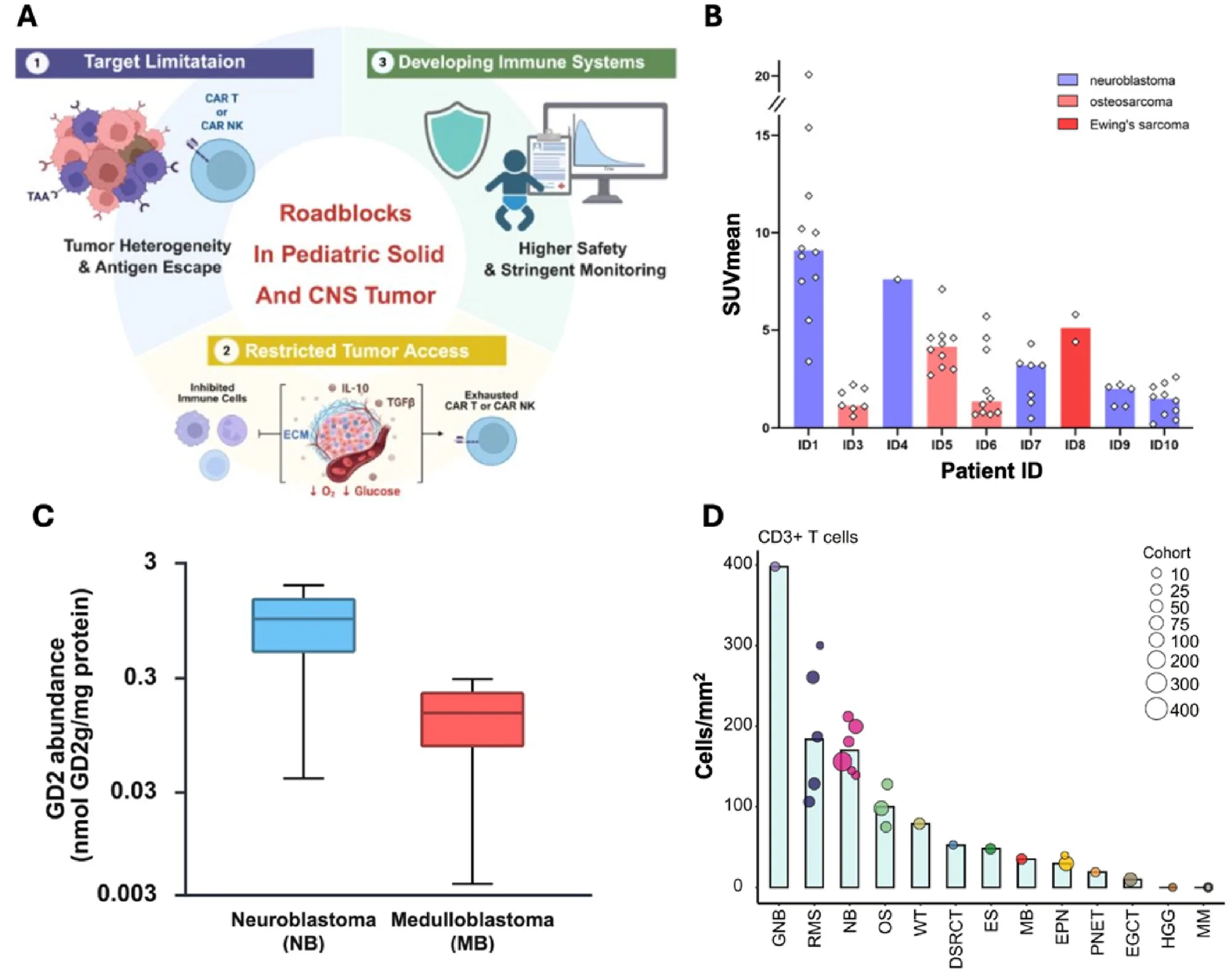
Quantifying biological and translational roadblocks in pediatric solid tumors. (A) Three interdependent barriers to engineered cell efficacy: target heterogeneity, physical and immunological tumor exclusion, and pediatric-specific safety constraints. Created in https://BioRender.com. (B) GD2 heterogeneity assessed by [55] Cu-GD2 PET/MRI in nine pediatric patients; columns indicate median standardized uptake value (SUV) mean per patient (up to 10 lesions per organ). Reproduced from [56]. CC BY 4.0. (C) GD2 abundance by LC-MS/MS in neuroblastoma (NB) and medulloblastoma (MB), shown as median with range (NB 0.54, MB 0.032 nmol mg^–1^ protein). Based on Paret *et al*, Cancers 2022. (D) Weighted-mean CD3+ T cell infiltration across pediatric (extra)cranial solid tumors, indicating a low immune baseline. Abbreviations are as follows: GNB, ganglioneuroblastoma; RMS, rhabdomyosarcoma; NB, neuroblastoma; OS, osteosarcoma; WT, Wilms tumor; DSRCT, desmoplastic small round cell tumors; ES, Ewing sarcoma; MB, medulloblastoma; EPN, ependymoma; PNET, primitive neuroectodermal tumors; EGCT, extra-cranial germ cell tumor; HGG, high-grade glioma; MM, multiple myeloma. Reprinted from [59], Copyright (2025), with permission from Elsevier.

Despite early clinical feasibility of CAR T and CAR NK cell therapies in pediatric solid tumors, durable benefit remains inconsistent. Importantly, many of the barriers observed in children are not simply scaled-down versions of adult challenges but reflect pediatric-specific biological, developmental, and safety constraints that shape how engineered cell therapies must be designed and evaluated. Across disease types and anatomic locations, three recurring design challenges emerge: 1) target limitation driven by heterogeneous and developmentally regulated antigen expression; 2) restricted tumor access and functional suppression within immune-excluded and anatomically constrained environments, particularly in the CNS; and 3) a higher safety and monitoring bar, particularly for the pediatric patients who are developing their immune systems together with these adoptively transferred cells (figure 2(A)) [12–14, 31, 41–43, 48–54]. We address each of these challenges in turn, and then consider in section 3.4 the engineering strategies that respond to them, together with the pediatric-specific limitations that accompany each.

### 3.1. Heterogeneous and developmentally dynamic antigen expression

Antigen heterogeneity is a significant obstacle in solid tumors across all age groups, not necessarily in pediatric patients only, but in children it carries additional complexity related to developmental biology and treatment history. Surface antigen density varies within individual lesions, between metastatic sites, and across time under therapeutic pressure. Even commonly pursued pediatric targets such as GD2 or HER2 exhibit mosaic patterns of expression, and prior therapy may reduce antigen density below activation thresholds for engineered T or NK cells. Quantitative imaging supports these findings in children. For example, clinical [55] Cu-GD2 PET/MRI demonstrates within-patient intratumoral variability (e.g. some lesions SUVmax >10 and high tumor-to-background) while others appear faint or negative (figure 2(B)) [56]. Proteomic measurements similarly show substantial differences in baseline protein expression between tumors; liquid chromatography-tandem mass spectrometry (LC–MS/MS) analyses likewise showed between-tumor gaps of GD2 abundance in neuroblastoma and medulloblastoma (e.g. neuroblastoma median 0.54 nmol mg^–1^ vs medulloblastoma 0.032 nmol mg^–1^) (figure 2(C)) [57]. Together, these explanations shed light on why single-antigen approaches may fail to cover the entire lesion and why relapse can occur through antigen-low or antigen-negative escape.

Beyond tumor heterogeneity itself, pediatric targeting must account for the narrow safety margin imposed by developing tissues, where low-level antigen expression in normal organs may carry greater long-term consequences than in adults. As a result, engineered cell products for children may need to tolerate antigen diversity rather than rely on uniform expression. Framing antigen diversity as a pediatric design constraint highlights the need for adaptive engineering approaches rather than single-antigen solutions. In this context, pediatric engineered cell products must be designed to accommodate antigen diversity rather than assume uniform expression across lesions or developmental stages. Importantly, because antigen expression can evolve during treatment, longitudinal *in vivo* measurement of target engagement may be necessary to guide adaptive dosing and inform next-generation construct design. The specific receptor designs implied here, including dual targeting and logic-gated or context-dependent sensing, will be discussed in later subsection.

### 3.2. Immune exclusion and anatomically constrained delivery

Immune exclusion is another major barrier that takes on distinct features in pediatric solid tumors. Many of these tumors display immune-cold or immune-excluded phenotypes at baseline, limiting endogenous priming and reducing the likelihood that CAR T or CAR NK cells will enter and remain functional without additional support. Pediatric TMEs frequently exhibit low danger signaling, myeloid- or Treg-skewed niches, and suppressive cytokine pathways such as TGF-β or adenosine, all of which attenuate effector function after tumor entry [58]. Physical barriers further limit trafficking, including dense extracellular matrix, aberrant vasculature, and, in the CNS, limited penetration across the blood brain barrier and restricted diffusion through tumor and peritumoral tissue. Systemic profiling across pediatric solid tumors supports this: integrated immunohistochemistry (IHC) shows low baseline infiltration (e.g. CD3+ median ≈48 cells mm^–2^, range 0–398), consistent with an immune-cold baseline (figure 2(D)) [59]. Under these conditions, TME modulation (e.g. suppressive-axis blockade, matrix remodeling) and delivery refinement (e.g. chemokine matching, locoregional/ICV dosing) are required and these examples will be discussed in section 3.4.

In pediatric CNS tumors, these constraints are often addressed operationally through delivery strategies such as intraventricular or intratumoral administration to reduce reliance on systemic trafficking. However, bypassing an anatomic barrier does not eliminate microenvironment-driven dysfunction, underscoring the need for tools that can distinguish insufficient delivery from post-entry functional suppression. These considerations suggest that improving efficacy in pediatric solid tumors will require simultaneous optimization of delivery route, microenvironmental resistance, and functional persistence. However, conventional response metrics provide limited insight into whether treatment failure reflects poor trafficking, inadequate activation, or rapid dysfunction after tumor entry. Quantitative tracking and functional imaging approaches therefore emerge as critical tools to disentangle these mechanisms and enable rational iteration of engineered cell therapies in anatomically complex pediatric diseases.

### 3.3. Heightened safety and monitoring requirements in a developing host

A final, distinctly pediatric roadblock arises from the long-term safety expectations associated with treating children who may live for decades after therapy. CAR T or CAR NK cells can persist long-term, which further highlights the importance of controllability and longitudinal monitoring. Acute toxicities such as CRS or neurologic events are already recognized risks for T cell therapies specifically, but in pediatrics, the acceptable threshold is lower, particularly for CNS-directed interventions. For example, a study reported the use of flotetuzumab to create a stable immunologic synapse between T cells (specifically by binding to CD3) and CD123+ acute myeloid leukemia in a phase 1 clinical trial in children and young adults. The results showed overall safety at 500 ng kg^–1^ d^–1^ and response rate of 20%, although CRS and capillary leak were frequent and generally early, and rare delayed inflammatory or neurologic toxicities remain possible, supporting longitudinal monitoring [60].

Real-time monitoring strategies that report biodistribution, persistence, and activation state may further support safer implementation by allowing early detection of off-tumor effects or excessive immune activation. Especially, together with some existing engineering solutions in pediatric solid tumor applications in the following subsection (section 3.4), as well as more in-depth discussion about the role of imaging (section 4), engineered cell therapies can be dynamically adjusted to maintain efficacy while minimizing long-term risk in children.

### 3.4. Bioengineering solutions and their pediatric constraints

The three aforementioned roadblocks motivate distinct engineering responses, each of which introduces a pediatric-specific limitation rather than a clean solution. First, to address the antigen heterogeneity, engineered cell products for children may need to tolerate antigen diversity rather than rely on uniform expression, motivating strategies such as dual targeting, logic-gated activation, or context-dependent sensing, including synthetic Notch (synNotch) circuits that incorporate environmental cues such as pH or cytokine gradients [61]. The relevance of this approach to pediatric CNS disease is illustrated by synNotch prime-and-kill circuits: when primed by a tumor-restricted or CNS-restricted antigen such as EGFRvIII or myelin oligodendrocyte glycoprotein, these circuits induced controlled CAR expression and achieved durable control of intracerebral patient-derived glioblastoma xenografts with heterogeneous antigen expression, without off-tumor killing and while preserving a less-exhausted, memory-enriched T cell state [62]. In children, however, the dependence of these circuits on coincident or sequential antigen recognition is harder to satisfy given mosaic, developmentally regulated antigen patterns, and the inducible expression that confers specificity can introduce delayed activation that may weaken early antitumor responses.

To overcome the suppressive TME, engineering approaches that introduce autocrine cytokine loops or enhance resistance to suppressive signals have shown promise in preclinical models. For example, a chimeric GM-CSF/IL-18 receptor, which converts a GM-CSF signal into IL-18 pathway signaling within the cell, sustained CAR T cell function and produced tumor regression in osteosarcoma and Ewing sarcoma models at cell doses where conventional CAR T cells were ineffective [63]. The same cytokine augmentation that drives this benefit, however, raises the risk of cytokine-mediated neurotoxicity and systemic inflammation in a developing host, whose tolerated inflammatory threshold is lower and in whom CNS inflammation can carry long-term cognitive consequences, which argues for cytokine activity that is tumor-localized or inducible rather than constitutive.

To satisfy the heightened safety bar, engineering solutions are increasingly focused on safety architectures, including drug-inducible suicide switches such as inducible caspase-9, which allow rapid ablation of transferred cells when necessary [64]. In pediatric settings, these control modules are not merely safeguards but integral components of therapy design, enabling clinicians to modulate activity as the child grows and recovers from therapy, and the value of such control is not only theoretical, as a safety switch was successfully activated in a child who developed severe toxicity during GD2-directed CAR T cell therapy [28]. Reversible pharmacologic control, which transiently pauses engineered cells rather than eliminating them, offers a graded alternative that may suit children well, because the optimal balance between sustained antitumor pressure and developmental safety can shift over months to years [55]. What remains insufficiently defined is when these systems should be activated, how rapidly they act, and how partial ablation affects efficacy, particularly in patients who may carry the construct for prolonged periods. Together, safety architecture and longitudinal tracking define an approach in which engineered cell therapies can be dynamically adjusted to maintain efficacy while minimizing long-term risk in children. Across all three responses, strategies validated in adult or hematologic disease cannot be assumed to perform equivalently in children, so combinatorial targeting, microenvironmental resistance, and controllable safety are best optimized together against the antigen biology, neurodevelopmental vulnerability, and extended survivorship that distinguish pediatric solid tumors [65].

Taken together, these pediatric-specific constraints highlight that durable success with engineered T cell or NK cell therapies will depend not only on improved receptor design but also on the ability to measure and refine therapeutic performance *in vivo*. Antigen heterogeneity, immune-excluded anatomy, and stringent safety requirements all introduce uncertainty regarding where transferred cells travel, how long they persist, and whether they remain functionally active after reaching the tumor. As a result, next-generation pediatric cell therapies may need to be developed alongside quantitative imaging and bioengineering tools that enable real-time assessment of delivery, engagement, and toxicity, shifting the paradigm from static infusion-based treatments toward dynamically monitored and iteratively optimized therapeutic systems.

## 4. Roles of imaging and measurement-enabled bioengineering for pediatric cell therapy

Given the pediatric-specific design constraints outlined above, a central opportunity in pediatric solid tumors is to treat engineered cell therapies as programmable biologics whose *in vivo* behavior can be quantitatively measured and iteratively refined, rather than as static, one-time infusions evaluated only by delayed radiographic response. This paradigm is particularly relevant in children, where tolerance for irreversible toxicity is low, especially in the CNS where inflammation, edema, and neurocognitive sequelae can carry long-term consequences. Accordingly, next-generation pediatric cell therapies should be developed alongside quantitative tools capable of reporting key *in vivo* parameters, including delivery to the tumor bed, persistence over time, spatial distribution across relevant compartments, and early evidence of tumor engagement. Such an approach supports rational decisions regarding route, schedule, and engineering design, to enable rational selection of route, schedule, and construct design.

Non-invasive imaging and tracking can address several pediatric-specific needs simultaneously. First, it can confirm whether infused cells reach target sites, which is a key uncertainty in immune excluded solid tumors and anatomically constrained CNS context. Second, it can quantify biodistribution and persistence to guide dosing and route selection, including evaluation of systemic vs. locoregional administration. Third, it can identify off-tumor accumulation patterns that may precede toxicity, which is particularly important in pediatrics where low level on-target, off-tumor recognition may have outsized developmental consequences. These advantages are amplified in pediatric tumors, where repeated tumor biopsy is rarely feasible and where conventional imaging can be difficult to interpret after immunotherapy. Because no single imaging modality can simultaneously provide high sensitivity, high spatial resolution, whole-body coverage, functional readout, and low-burden repeatability, modality selection should be guided by the specific biological question being asked and by pediatric feasibility considerations (table 3).

**Table 3.** Imaging modalities for monitoring pediatric engineered cell therapies.

Imaging modality	Readout	Pros	Cons	Notes
PET/SPECT	• Biodistribution • Functional probe uptake • Radioisotope-labeled cells	• No depth limitation • High sensitivity • Whole body imaging	• Ionizing radiation • Low spatial resolution • High cost	Consider cumulative radiation exposure for pediatric applications

MRI	• Anatomical response • Edema • Perfusion • T1/T2 contrast agent-labeled cells	• No depth limitation • High resolution • Whole body imaging • Soft-tissue and CNS imaging • Non-ionizing radiation	• Lower sensitivity • Low temporal resolution • High cost	Longitudinal imaging could be challenging due to scan duration and anesthesia burden

Ultrasound/photoacoustic imaging	• Anatomial response • Perfusion • Oxygenation • Optical absorber-labeled cells	• High spatial and temporal resolution • Non-ionizing radiation • Multiplexing capabilities • Easier access	• Lower imaging depth over PET/MRI/CT	Attractive for pediatric applications but less suitable for CNS lesions

Optical imaging	• Bioluminescence • Fluorescence • Optical scatter; Fluorescence/luminescence-labeled cells	• High sensitivity • High spatial and temporal resolution • Multiplexing capabilities	• Very limited imaging depth (µm scale)	Most useful for intraoperative imaging

X-ray/CT	• Anatomical response • Bone structure • Metal/CaP-labeled cells	• No depth limitation • Whole body imaging • Easier access	• Ionizing radiation • Lower soft-tissue contrast	Consider cumulative radiation exposure for pediatric applications

**Figure 3.**
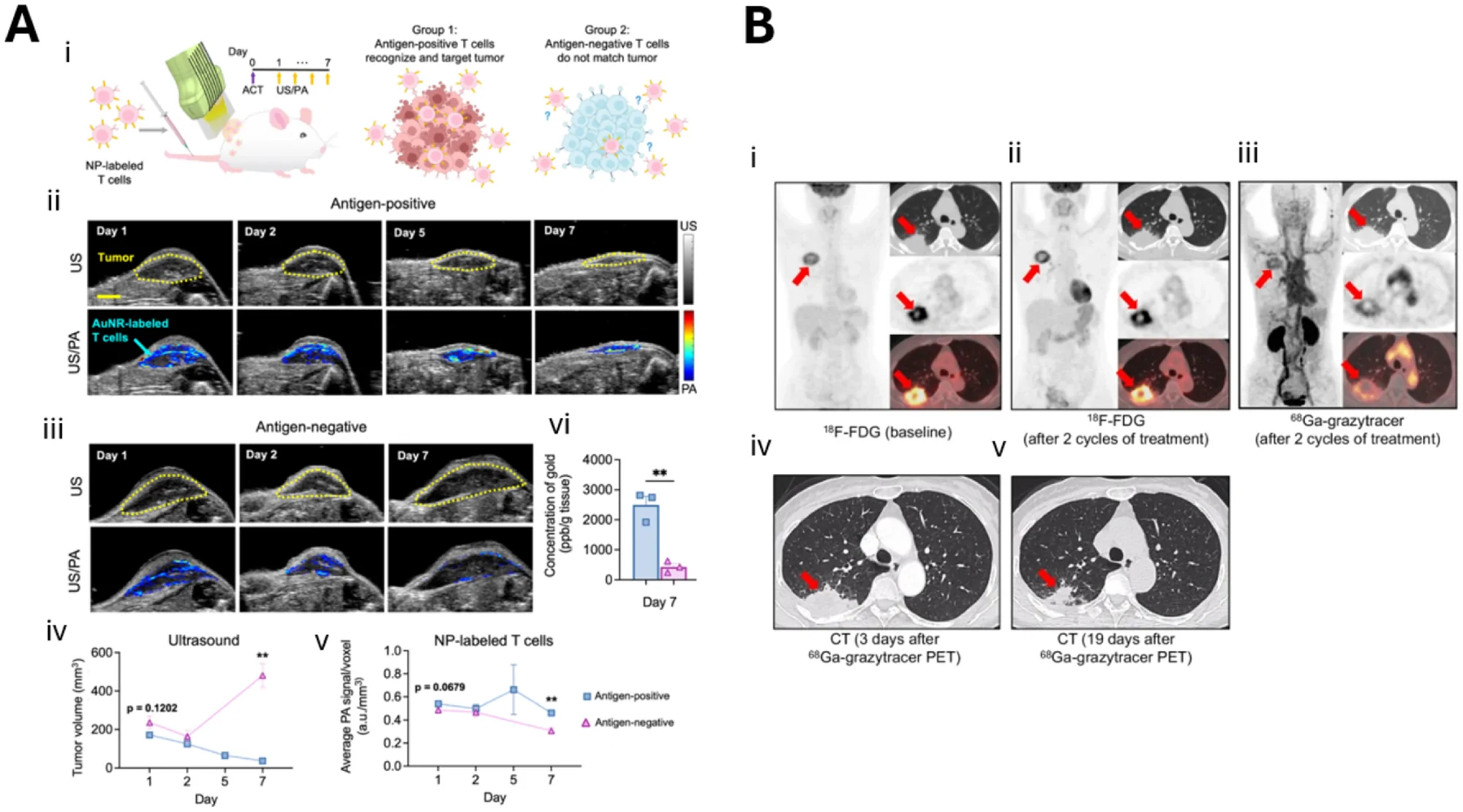
Quantitative imaging tools for monitoring engineered cell therapies *in vivo*, reporting delivery, spatial distribution, and early tumor engagement. (A) Ultrasound/photoacoustic (US/PA) imaging detects nanoparticle (NP)-labeled T cells at the primary tumor after adoptive transfer, enabling longitudinal quantification of tumor volume (3D US) and T cell accumulation (3D PA). Reprinted with permission from [68]. Copyright (2025) American Chemical Society. (B) [68] Ga-grazytracer (granzyme B) PET distinguishes pseudoprogression from true response in a patient with lung adenocarcinoma, where unchanged tumor size with rising [18] F-FDG uptake after immuno-chemotherapy was clarified by grazytracer PET and confirmed by subsequent tumor shrinkage on CT. Arrows indicate tumors. Reproduced from [74], with permission from Springer Nature.

Research endeavors are ongoing specifically focused on developing and applying non-invasive, *in vivo* imaging strategies to track engineered immune cells and to quantify their spatiotemporal behavior within solid TMEs, while not necessarily limited to pediatric cancer scenario, with the goal of linking cell distribution to therapeutic response and safety [66–68]. Practically, tracking approaches can be divided into complementary categories: biodistribution and persistence can be assessed using PET-based methods, including direct radiolabeling for early trafficking or reporter gene systems for longitudinal monitoring [69, 70]. Because minimizing cumulative radiation exposure is a practical priority in children, radiation-free modalities are also attractive [71, 72]. Ultrasound provides real-time, bedside-compatible anatomic imaging, and photoacoustic imaging adds molecular contrast to map exogenous absorbers and functional tissue features for immune cell tracking in solid TME (figure 3(A)) [68]. In parallel, nanoparticle-engineered CAR T cell imaging platforms have also been developed for non-invasive tracking with MRI, supporting a broader multimodal paradigm (figure 3(B)) [73]. Although most cell-tracking platforms have been developed outside pediatric solid tumors, the same strategies are technically transferable, with pediatrics-specific constraints (radiation exposure, sedation burden, and limited biopsy access).

Equally important is functional assessment of what the cells are doing after arrival. In pediatric solid tumors, lack of response can reflect poor trafficking, antigen heterogeneity, or microenvironment-driven dysfunction. Functional imaging biomarkers, including emerging PET strategies that report effector activation, can help distinguish delivered-but-dysfunctional cells from failed delivery and can improve interpretation of post-treatment changes in tumors where inflammation can confound standard imaging [74–76]. Especially, imaging strategies for pediatric engineered cell therapy should be selected according to whether the goal is to measure systemic biodistribution, local tumor delivery, functional engagement, or treatment-associated toxicity. In children, the value of each modality depends not only on technical performance, but also on whether it can be repeated safely and practically over time. Together, measurement-enabled development provides a path to move beyond binary response assessments toward mechanism-informed iteration of engineered cell therapies in pediatric solid malignancies.

## 5. Summary and translational outlook

Taken together, the challenges and opportunities discussed here support a pediatric-first framework for engineered cellular therapies in solid tumors. In this framework, CAR T and CAR NK products are designed as controllable systems that must 1) tolerate heterogeneous and developmentally dynamic antigen expression, 2) function within immune-excluded and suppressive microenvironments, and 3) meet a higher safety bar appropriate for a developing host. These requirements motivate multi-antigen and logic-gated targeting strategies, microenvironment-resistance designs, and built-in safety architectures (e.g. inducible switches) as core components rather than optional add-ons. Critically, quantitative *in vivo* measurement becomes part of the therapeutic design loop, enabling discrimination among inadequate trafficking, antigen escape, and post-entry dysfunction and supporting rational iteration of route, dosing schedule, and construct architecture. In pediatrics, efficacy, safety, durability, manufacturability, and access are interdependent; designs that neglect any one of these dimensions often fail to translate.

From clinical standpoint, translating this strategy into practice requires parallel evolution in trial design, therapeutic evaluation, and integration within pediatric oncology care. Patient selection may increasingly rely on quantitative assessment of target expression and spatial heterogeneity, incorporating molecular profiling and functional imaging to identify children most likely to derive benefit while limiting exposure in antigen-low disease. Longitudinal monitoring of biodistribution and activation can inform adaptive treatment strategies, including decisions regarding repeat locoregional administration, timely incorporation of combination approaches when trafficking or engagement is insufficient, and improved differentiation of true progression from treatment-related changes. Embedding controllability features within engineered cell products provides clinicians with actionable safeguards in the event of neurotoxicity or systemic inflammatory syndromes and may broaden eligibility to patients otherwise excluded due to safety concerns. Importantly, clinical success in this context should extend beyond conventional response rates to encompass durable remission without cumulative neurocognitive decline, preservation of organ function, feasibility of manufacturing within disease-relevant timelines, and equitable access across institutions. By explicitly linking engineering innovation to clinically actionable endpoints and survivorship outcomes, this pediatric-centered framework seeks to ensure that advances in cellular engineering and imaging translate into measurable, sustained improvements in both survival and long-term quality of life for children.

## Data Availability

No new data were created or analyzed in this study.
